# A UNIQUE APPROACH TO INTERPROFESSIONAL TRAINING USING THE ANNUAL WELLNESS VISIT HEALTH RISK ASSESSMENT

**DOI:** 10.1093/geroni/igz038.1448

**Published:** 2019-11-08

**Authors:** Anna Faul

**Affiliations:** University of Louisville, Louisville, Kentucky, United States

## Abstract

The Kentucky (KY) Rural & Underserved Geriatric Interprofessional Education Program (KRUGIEP) participated in a unique innovative approach to the implementation of the Medicare Annual Wellness visit in collaboration with the Dartmouth GWEP. The model focused on the integration of students into the process of conducting the Health Risk Assessment through community based home visits. This talk will focus on this unique program and the participation in a multi-GWEP learning collaborative.

